# Shegan Mahuang Decoction May Reduce Airway Inflammation in Neutrophilic Asthmatic Mice by Improving the Mitochondrial Function of Bronchoalveolar Lavage Fluid Exosomes

**DOI:** 10.1155/2022/2477510

**Published:** 2022-12-19

**Authors:** Hui-na Lu, Zhou Fu, Xia Chen, Ming-ming Yang, Yun-fang Chen, Li-li Yang

**Affiliations:** ^1^Department of Respiratory Medicine, Ministry of Education Key Laboratory of Child Development and Disorders, National Clinical Research Center for Child Health and Disorders, China International Science and Technology Cooperation Base of Child Development and Critical Disorders, Children's Hospital of Chongqing Medical University, Chongqing, Chongqing Key Laboratory of Pediatrics, China; ^2^Department of Pediatrics, Chongqing Hospital of Traditional Chinese Medicine, Chongqing, China; ^3^Department of Pediatrics, 958 Hospital of Army PLA, Chongqing, China

## Abstract

Asthma is a common pulmonary disease mainly caused by the infiltration of neutrophils. There is a limit to the therapeutic effects of the available asthma drugs on neutrophilic asthma. Shegan Mahuang Decoction (SMD) is one of the representative traditional Chinese medicine (TCM) prescriptions for asthma, and it can effectively relieve the clinical symptoms of patients. However, the effect of SMD on the treatment of neutrophilic asthma remains unknown. In this study, a mouse model of neutrophilic asthma induced by lipopolysaccharide (LPS)/ovalbumin (OVA) was established, and the effect of a modified SMD prescription on the model was evaluated. After treatment, SMD was demonstrated to be therapeutically effective on asthmatic mice via airway resistance detection and lung pathology and was able to affect cytokine levels *in vivo*. Further experiments verified that SMD regulated the expression of mitochondrial function proteins in bronchoalveolar lavage fluid (BALF) exosomes. The results demonstrate that SMD confers a therapeutic effect on a neutrophilic asthma mouse model, and it may reduce neutrophil airway inflammation by regulating myeloid-derived regulatory cell (MDRC) function and airway exosome mitochondrial function.

## 1. Introduction

As described by the Global Initiative for Asthma (GINA), asthma is a complex heterogeneous disease characterized by chronic airway inflammation and airway hyperresponsiveness [1]. At present, asthma has become the second most prevalent chronic lung disease in the world, and it severely threatens the health and quality of life of the public because of its high incidence rate and poor control rate. Therefore, there is an urgent need to determine the pathological mechanism of asthma so that more effective treatments can be developed.

Asthma is generally considered to be a type I allergic airway disease mediated by T helper 2 (Th2 cells) and immunoglobulin E (IgE), and it is usually accompanied by a dramatic increase in airway and lung eosinophils [2]. As reported by recent studies, in approximately 50% of asthma cases, especially moderate to severe asthma and glucocorticoid-resistant asthma, a significant increase in neutrophils is measured [3, 4].

It has been demonstrated that myeloid-derived regulatory cells (MDRCs) regulate airway inflammation by producing free radicals (nitric oxide and superoxide) that can influence the function of T cells [5]. Apart from producing free radicals, a recent study found that MDRCs may secrete exosomes that contain pro and anti-inflammatory mediators to modulate the progress of asthma [6]. In addition to conventional immune modulation mechanisms, exosomes secreted by bronchoalveolar lavage fluid (BALF)-derived MDRCs were able to carry functional mitochondria to T cells and subsequently modulate the immune activation or dampening of asthma [7].

Shegan Mahuang Decoction (SMD) is a representative prescription of traditional Chinese medicine (TCM), and it can effectively relieve the clinical symptoms of asthma patients [8]. Although studies have claimed that SMD may take effect by regulating the activity of immune cells, the underlying mechanism remains unclear. Hence, in order to explore the possible mechanism, the present study evaluated the therapeutic effect of a modified SMD prescription on a neutrophilic asthma mouse model. The histopathology results indicated that the modified SMD may attenuate asthmatic symptoms, and the probable reason was that SMD improved the mitochondrial function of the exosomes in the BALF.

## 2. Materials and Methods

### 2.1. Mice

A total of 48 female 6-week-old SPF-grade BALB/c mice were obtained from Chongqing Enswell Biotechnology Co. Ltd. and randomly divided into a negative control (NC) group, neutrophilic asthma model (NA) group, SMD low-dose (SMD-L) group, and SMD high-dose (SMD-H) group (*n* = 12). The mice were housed using a 12-hour light and dark cycle with ad libitum access to food and clean water. All procedures were performed in accordance with the World Medical Association Declaration of Helsinki. All animal protocols were approved by the Animal Ethics and Welfare Committee of Chongqing Hospital of Traditional Chinese Medicine (approval number: 2020-DWSY2-LHN).

### 2.2. Preparation of SMD

For the preparation of SMD, the following components were used: 5 g of Honey ephedra, 6 g of Shegan, 1 g of Asarum, 3 g of Schisandra, 6 g of Wine scutellariae, 6 g of Aster, 6 g of Radix Peucedani, 5 g of Fried semen lepidii, and 2 g of Baked licorice, for a total of nine herbs as shown in Table 1. Then, fresh water six times the weight of the herbs was added to the herbs, which were decocted for 60 minutes, and the decoction was repeated twice before filtering. The filtrate was concentrated and dried in a vacuum oven to obtain a dry extract, which was finally crushed to fine powder. For usage, the SMD decoction for the SMD-L group was prepared by dissolving SMD powder in fresh water to a concentration of 0.6 g/mL, and the concentration used for the SMD-H group was 1.2 g/mL.

### 2.3. Establishment of a Neutrophilic Asthma Mouse Model

Preparation of lipopolysaccharide (LPS) and ovalbumin (OVA) stock solution: 0.03 g LPS powder was dissolved in 0.3 mL of normal saline to make a 100 *μ*g/*μ*L solution. For usage, this solution was diluted 10 times to 10 *μ*g/*μ*L and stored at 4°C. For OVA, 0.1 g of powder was dissolved in 1 mL of normal saline to make a 100 *μ*g/*μ*L solution. For usage, this solution was diluted 100 times to 1 *μ*g/*μ*L and stored at 4°C.

BALB/c mice in the NA and SMD treatment groups were anesthetized on day 1 and day 7; LPS (4 *μ*g/g, calculated based on mouse body weight) was instilled from the airway, and OVA (100 *μ*L/mice) was injected intraperitoneally. From the 14th day to the 18th day, the mice were challenged by aerosolizing 5 mL of OVA solution every day, thereby establishing a neutrophilic asthma mouse model [9]. The mice in the control group were sensitized with phosphate-buffered saline (PBS) instead of LPS or OVA, challenged with normal saline, and the experiment was performed synchronously with the model group.

**Figure 1:**
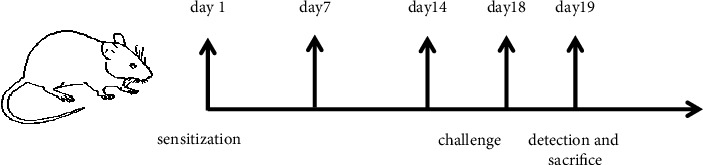
Schematic diagram of the experiment. On day 1 and 7, the mice were sensitized with LPS and OVA in the NA group and SMD treatment groups, while the NC group was sensitized with PBS. On days 14 to 18, all mice were challenged by OVA (except for the control mice, which received saline solution). Then, saline solution was administered to the NC and NA groups, and the SMD-L and SMD-H groups were treated with SMD, twice a day. On day 19, the mice were anesthetized, and samples were harvested for analysis.

After model establishment, intragastric administration of drugs was performed for the mice in each group, and the same amount of normal saline (10 *μ*L/g) was intragastrically administered to the mice in the NC and NA groups. The SMD-L and SMD-H groups were treated with relative SMD, for 5 consecutive days, twice a day. All mice were euthanized for analysis 24 hours after establishing the model (Figure 1).

### 2.4. Obtaining BALF

After the mice were anesthetized with pentobarbital sodium, the throat skin was cut open to expose the trachea, and an indwelling needle with a needle tube fastened with sutures was inserted, followed by pulling out the hard needle. Next, 0.5 mL precooled PBS was injected into the lung, and then, four lavages were conducted. The BALF extracted from each group (approximately 18 mL) was mixed into one tube. Cell pellets were obtained for subsequent analysis by centrifuging the BALF at 1000 ×g (10 min, 4°C). Finally, the mice were euthanized by cervical dislocation.

### 2.5. Airway Responsiveness Assessment and Lung Histopathology

After anesthetizing with pentobarbital sodium, the tracheae of the mice were fully exposed, and tracheal intubation was performed to connect the small animal ventilator system to examine the airway resistance of the mice. Briefly, changes in airway resistance were firstly measured in the basal state, and then inhalation with different concentrations of methacholine (0 mg/mL, 3.125 mg/mL, 6.25 mg/mL, 12.5 mg/mL, 25 mg/mL, and 50 mg/mL) to stimulate an asthma attack was carried out. After that, the mice were euthanized by cervical dislocation.

For lung histopathology, mice were euthanized by carbon dioxide asphyxia. Lung lobes were extracted and fixed with 4% paraformaldehyde for 24 h and then embedded in paraffin. The tissues were cut into 5 *μ*m thick sections, deparaffinized, hydrolyzed, and stained with hematoxylin and eosin (H&E).

### 2.6. Enzyme-Linked Immunosorbent Assay (ELISA) for Cytokines

After thoroughly mixing, the obtained BALF was centrifuged at 1800 rpm, and the supernatant was used to detect the change in cytokine levels (IL-4, IL-13, IL-33, and TGF-*β*) using an ELISA kit (Neobioscience, China), according to the manufacturer's instructions.

### 2.7. Extraction of Exosomes

Exosomes were purified from BALF using the differential centrifugation method. In brief, 80 mL of BALF was centrifuged at 300 ×g for 10 min at 4°C to remove cells, and the supernatant was further centrifuged at 2000 ×g for 10 min at 4°C to remove apoptosis corpuscles. The supernatant was centrifuged again at 10,000 ×g for 30 min at 4°C and passed through a 0.2 *μ*m cellulose acetate filter, and then, the filtrate was centrifuged at 100,000 ×g for 70 min at 4°C. The resulting pellet was washed with fresh PBS, centrifuged at 100,000 ×g for 70 min at 4°C, and resuspended in 200 *μ*L of fresh sterile PBS.

### 2.8. Western Blot

The extracted exosomes were lysed in radioimmunoprecipitation assay (RIPA) buffer and then centrifuged at 14,000 rpm for 30 min at 4°C. Lysate proteins were loaded and electrophoresed on 12% polyacrylamide gels and then transferred to polyvinylidene fluoride (PVDF) membranes by electroblotting at 80 V for 120 min. Membranes were blocked in 5% non-fat milk and Tris-buffered saline (TBS) containing 0.1% Tween-20 (TBST) for 1 hour at room temperature, incubated with primary antibody overnight at 4°C, and washed in TBST. The membrane was then incubated with horseradish peroxidase (HRP)-conjugated secondary antibody for 1 hour at room temperature. The target protein was detected using an enhanced chemiluminescence solution with X-ray film.

### 2.9. Transmission Electron Microscopy (TEM) Characterization

For TEM analysis, exosomes were placed on copper and treated with phosphotungstic acid for negative staining. A JEM-1400PLUS transmission electron microscope (Japan Electron Optics Laboratory) was used to view the morphology of the exosomes.

### 2.10. Fluorescence-Activated Cell Sorting (FACS) for MDRC

The BALF cells obtained above were incubated with PE-Cy7-CD11c, FITC-A-HLA-DR, and APC-CD11b antibodies (antibodies were diluted with staining buffer to an appropriate concentration) and then resuspended for cell sorting by a BD FACSAria III flow cytometer.

### 2.11. Statistical Analysis

The statistical analysis was performed using GraphPad Prism software. The data are presented as mean ± standard deviation (SD). A *P* value less than 0.05 was considered to be statistically significant. Two-tailed unpaired Student's *t* test or analysis of variance (ANOVA) with Tukey post hoc test for data with more than two groups was performed to determine statistical significance.

## 3. Results

### 3.1. Inhibitory Effect of SMD on the Neutrophilic Asthma Mouse Model

**Figure 2:**
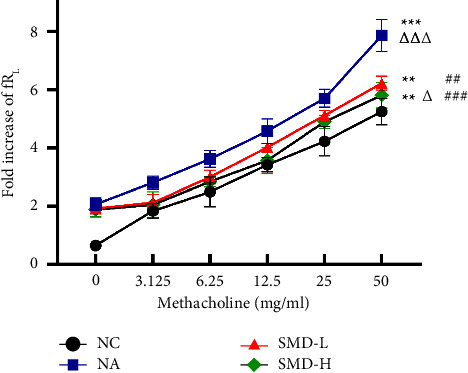
Changes in airway resistance after drug administration. The data are presented as mean ± SEM. All data are representative of four independent experiments with a minimum of three to five mice per group. ^*∗*^*P* < 0.05, ^*∗∗*^*P* < 0.01, and ^*∗∗∗*^*P* < 0.001 compared to the NC group; ^#^*P* < 0.05, ^##^*P* < 0.01, and ^###^*P* < 0.001 compared to the NA group; and ^Δ^*P* < 0.05, ^ΔΔ^*P* < 0.01, and ^ΔΔΔ^*P* < 0.001 compared to the SMD-L group.

After successful modeling, the corresponding SMD dosages were administered to the mice in the four experimental groups. Subsequently, the airway resistance of the mice in each group was measured using a small animal pulmonary function measurement system. The airway resistance to methacholine increased with the increase in the inhaled concentration in the control group of mice. Compared with the NC group, the airway resistance was significantly increased in the NA group (Figure 2; *P* < 0.001). Compared with the NA group, an evident elevation in *R*_L_ was observed in the SMD groups (Figure 2; *P* < 0.01), and there was lower airway resistance in the SMD-H group as compared to the SMD-L group (Figure 2; *P* < 0.05).

**Figure 3:**
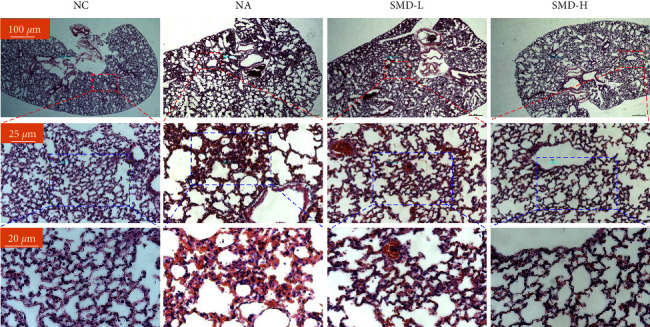
H&E staining results show the therapeutic effects of SMD on the lung tissue of the neutrophilic asthmatic mice. Red and blue rectangles indicate magnified views.

The H&E staining results showed that the bronchial structure of the lung tissue in the NC group was complete, the alveolar structure was clear, and the surrounding inflammatory cells were less infiltrated. For the NA group, the bronchial lumen of the lung tissue was narrow, and the alveolar and lung tissue structure was destroyed. Epithelial cells were shed, exfoliated cells were present in the alveolar cavity, and infiltration of a large number of inflammatory cells occurred around the bronchi and blood vessels. Compared with the NA group, in the SMD-L and SMD-H groups, there was significantly less damage to the lung tissue structure, and less bronchial mucosa edema and inflammatory cell infiltration, with clearly more normalized features in the SMD-H group, as shown in Figure 3.

### 3.2. SMD Attenuated the Expression of Cytokines in Neutrophilic Asthmatic Mice

**Figure 4:**
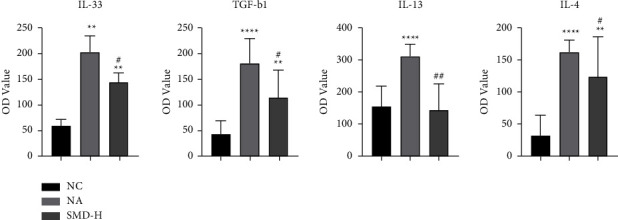
Effects of SMD on cytokine secretion in BALF. The expression levels of IL-33, TGF-b1, IL-13, and IL-4 were measured with ELISA. The data are presented as mean ± SD. All data are representative of three independent experiments with a minimum of three to five mice per group. ^*∗*^*P* < 0.05, ^*∗∗*^*P* < 0.01, ^*∗∗∗*^*P* < 0.001, and ^*∗∗∗∗*^*P* < 0.0001 compared to the NC group; ^#^*P* < 0.05, ^##^*P* < 0.01, and ^###^*P* < 0.001 compared to the NA group.

The pathological section results showed a stronger therapeutic effect by SMD-H on neutrophilic asthmatic mice. The changes in cytokines (IL-4, IL-13, IL-33, and TGF-*β*1) in the BALF of SMD-H group mice were subsequently detected using ELISA. As shown in Figure 4, compared with the NC group, the expression of IL-33 (*P* < 0.01), TGF-*β*1 (*P* < 0.0001), IL-13 (*P* < 0.0001), and IL-4 (*P* < 0.0001) in the BALF was significantly increased in the NA group, while only IL-33 (*P* < 0.01), TGF-*β*1 (*P* < 0.01), and IL-4 (*P* < 0.01) were significantly increased in the SMD-H group. However, when comparing the SMD-H group with the NA group, IL-33 (*P* < 0.05), TGF-*β*1 (*P* < 0.05), IL-13 (*P* < 0.01), and IL-4 (*P* < 0.05) significantly decreased in the SMD-H group.

### 3.3. SMD Regulated the Airway Exosomes in the BALF of Neutrophilic Asthmatic Mice

**Figure 5:**
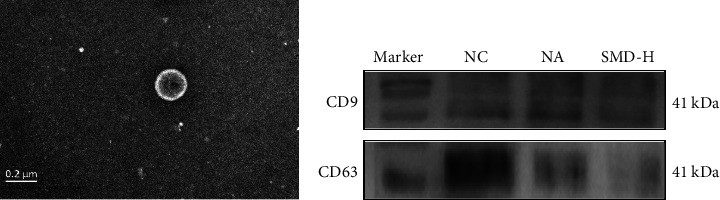
Characterization of the exosomes extracted from BALF. (a) The TEM results show the typical structure of exosomes. (b) Western blot analysis on the markers of exosomes.

Exosomes were extracted from the BALF and examined using TEM (Figure 5(a)). The results showed a typical double-membrane round vesicle structure with a particle size ranging from 30 to 150 nm in the obtained exosomes. Subsequently, the marker proteins of exosomes (CD9 and CD63) were measured using western blot (Figure 5(b)), and the results showed that CD63 was highly expressed in the exosomes of the control group, while CD63 expression was sharply decreased in the model group, and CD63 decreased further after SMD treatment. On the contrary, the expression of CD9 in the control group was lower than that in the model group and recovered after SMD treatment.

**Figure 6:**
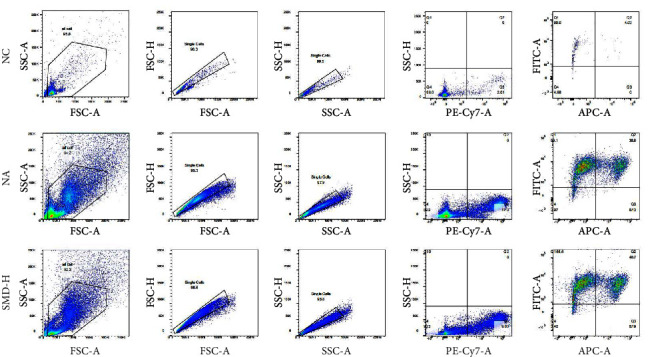
Effect of SMD treatment on the number of MDRCs in the BALF.

The difference in MDRCs in BALF was measured by flow cytometry. As shown in Figure 6, the proportion of MDRCs in the control group was only 0.1%, and the proportion increased to 6.6% in the asthma model group. After treatment with SMD, the proportion of MDRCs was reduced. Furthermore, the expression of mitochondrial fusion protein (MFN1), mitochondrial membrane protein (MIRO1), and mitochondrial transcription activator (NRF1) and the expression changes in mitophagy-related proteins LC3B and Beclin1 in the exosomes were measured using western blot.

**Figure 7:**
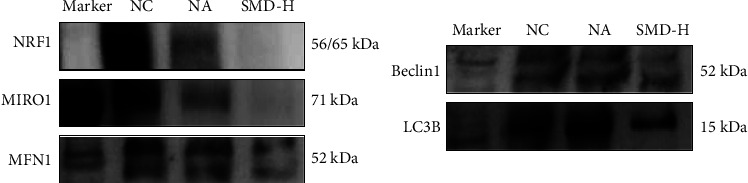
Western blot results for NRF1, MIRO1, MFN1, Beclin1, and LC3B expression in exosomes.

The results showed that the expression of MFN1, MIRO1, and NRF1 in the exosomes of the control group was higher than that of the NA group and the SMD-H group (Figure 7). However, the expression of mitophagy-related proteins LC3B and Beclin1 in the exosomes of the control group was relatively low but significantly increased in the NA group. After SMD treatment, the expression of LC3B and Beclin1 decreased, indicating that mitochondrial autophagy was activated in the asthma model, while it was inhibited by SMD treatment.

## 4. Discussion

Asthma is generally well managed by the inhalation of corticosteroids, but the long-term use of corticosteroids may cause side effects, and 40% of patients will ultimately fail to respond to corticosteroids [10]. With the development of medicinal botany, an increasing number of natural plant extracts have been reported to have therapeutical effects on a diverse array of diseases, such as neuroprotective properties [11, 12] and antioxidant properties [13, 14].

SMD was discovered by Zhang Zhongjing in the Eastern Han dynasty, and it is a classic TCM formula for treating asthma that is composed of belamcanda, ephedra, ginger, pinellia, aster, coltsfoot, schisandra, asarum, and jujube [15]. In China, SMD has been widely used to treat cough variant asthma, post-infection cough, bronchitis, and other airway diseases [16, 17]. Although studies revealed that SMD can attenuate asthma by regulating the expression of inflammatory mediators such as of INF-*γ*, IL-4, IL-10, IL-13, and TNF-*α* [18, 19], the exact mechanism underlying its protective effect remains unclear.

Based on the physique of children and the years of clinical experience of our group, we modified the classic SMD prescription to obtain a pediatric formula [20], and we used it in the present study to explore the possible mechanisms of how SMD affects asthma. In the theory of TMC, children with asthma always have a long course of disease, and phlegm-fluid retention and phlegm-qi stagnation would lead to a tendency towards fever. Thus, on the basis of ephedra, asarum, and other pungent ingredients, skullcap (*Scutellaria baicalensis* Georgi) and flixweed (*Descurainia sophia* L.) were added to our modified SMD prescription for clearing heat and descending qi, relieving asthma and cough, and balancing cold and heat, so as to cure existing diseases and prevent underlying diseases.

In this study, the modified SMD was administered to neutrophilic asthmatic mice to verify its therapeutic effect on asthma. The results showed that SMD relieved the symptoms of the mice, as the airway resistance significantly decreased, and the lung pathological results were significantly ameliorated. SMD was also found to attenuate inflammation by downregulating the expression of pro-inflammatory cytokines in BALF, including IL-4, IL-13, IL-33, and TGF-*β*1.

Previous studies reported that there are important immunomodulatory effects governed by the exosomes in the BALF of asthma patients [6, 21]. Exosomes are nanoscale membrane-enclosed structures released into the surrounding environment by different cells, and they contain functional RNAs, proteins, and lipids that can be shuttled from one cell to another, thereby affecting cellular activity [22, 23]. In addition to RNAs, proteins, and lipids, recent studies found that the mitochondria in exosomes also play an important role in modulating recipient bioenergetics [24, 25]. As a multifunctional organelle, mitochondria play key roles in biosynthetic reactions, ATP synthesis, and the modulation of immune system responses. Dysregulation of mitochondria-dependent signaling pathways may lead to physiological and pathophysiological consequences [26, 27]. Studies have reported that airway MDRCs can transfer mitochondria to T cells via exosomes and regulate T cell proliferation with the mitochondrial network of T cells, which in turn modulate airway responses in asthma models [7, 28].

We hypothesized that SMD attenuates asthma by regulating the function of MDRC-derived mitochondria, which are exosomally transferred to T cells. To verify this hypothesis, exosomes were isolated and extracted from the BALF of neutrophilic asthmatic mice. The western blot results showed that the expression of mitochondria-related proteins in the exosomes of SMD-treated asthmatic mice was significantly decreased, while mitophagy proteins were significantly increased.

Autophagy represents a series of cellular processes that aim to recycle components and organelles through channeling them into lysosomes, which protect cells from damaged cellular components, induced inflammation, or reactive oxygen species (ROS) [29, 30]. The clearance of damaged mitochondria (i.e., mitophagy) is a type of selective autophagy involving apoptotic signaling pathways [31, 32]. We speculated that the mitochondria were severely damaged in the asthma model group, and the administration of SMD activated mitophagy to clear the damaged mitochondria.

The present study confirmed the therapeutic effect of SMD on a mouse model of neutrophilic asthma. With the perspective of mechanism of action, SMD regulated the function of the mitochondria, which is encapsulated in exosomes, and the exosomes acted as a mediator for intercellular presentation. MDRCs were able to produce mitochondria-containing exosomes, and SMD attenuated the upregulation of MDRCs in the asthma model group. Therefore, we speculated that SMD might regulate the function of the mitochondria in exosomes produced by MDRCs and then regulate downstream T cells, thereby affecting the secretion of BALF cytokines in the mouse model of neutrophilic asthma and decreasing the occurrence of inflammation. Further study should verify whether the abovementioned exosomes are derived from MDRCs and more closely examine how they regulate the function of downstream T cells.

## 5. Conclusions

In this study, a mouse model of neutrophilic asthma was established using LPS/OVA. Then, the effect of SMD on neutrophilic asthmatic mice was evaluated, and it was demonstrated to be efficacious. The potential mechanism was SMD was a reduction of neutrophil airway inflammation by regulation of myeloid-derived regulatory cell (MDRC) function and airway exosome mitochondrial function.

## Figures and Tables

**Table 1:** Composition of herbs used for the preparation of Shegan Mahuang Decoction.

Name of herb	Dosage (g)
Ephedra (Ma Huang; honey Herba Ephedrae) *Ephedra sinica* Stapf.	5
Blackberry Lily rhizome (She Gan; Rhizoma Belamcandae) *Belamcanda chinensis* (L.) DC.	6
Manchurian Wild Ginger (Xi Xin; Herba Asari) *Asarum heterotropoides* L.	1
Schisandra berries (Wu Wei Zi; Fructus Schisandrae) *Schisandra chinensis* (Turcz.) Baill	3
Skullcap (Huang Qin; wine Herba Scutellariae) (*Scutellaria baicalensis* Georgi)	6
Tatarian Aster root (Zi Wan; Radix Asteris) *Aster tataricus* L.	6
Hongfennel root (Qian Hu; Radix Peucedani) *Peucedanum praeruptorum* Dunn	6
Flixweed/Tansy Mustard seeds (Ting Li Zi; fried Semen Lepidii) *Descurainia Sophia* L.	5
Licorice root (Gan Cao; baked Radix Glycyrrhizae) *Glycyrrhiza uralensis* Fisch. ex DC	2

## Data Availability

The data used to support the findings of this study are available from the corresponding author upon request.
